# Di­aqua­{μ_2_-*N*,*N*′-bis­[(cyclo­hexa­nyl­idene)amino]­oxamide}­bis­(tri­phenyl­phosphane)silver(I) dinitrate

**DOI:** 10.1107/S1600536813034454

**Published:** 2014-01-04

**Authors:** Ruthairat Nimthong, Nattakunya Thepsena, Walailak Puetpaiboon, Yupa Wattanakanjana

**Affiliations:** a Department of Chemistry, Faculty of Science, Prince of Songkla University, Hat Yai, Songkhla 90112, Thailand

## Abstract

The dinuclear title compound, [Ag_2_(C_14_H_22_N_4_O_2_)(C_18_H_15_P)_2_(H_2_O)_2_](NO_3_)_2_, lies across an inversion center and consists of two [Ag(H_2_O)(PPh_3_)] units bridged by a bis­(cyclo­hexa­none)oxalydihydrazone ligand. The charge-balance is supplied by two nitrate anions. The symmetry-unique Ag^I^ ion is in a distorted tetra­hedral geometry coordinated by a P atom from a tri­phenyl­phosphane ligand, an O atom from a water mol­ecule and a bis­(cyclo­hexa­none)oxalydihydrazone ligand bidentate chelating through the O atom and one of N atoms. In the crystal, O—H⋯O and N—H⋯O hydrogen bonds link the components, forming chains along the *b-*axis direction. These chains are connected through weak C—H⋯O hydrogen bonds, leading to the formation of a two-dimensional supra­molecular network parallel to (001).

## Related literature   

For potential applications of hydrazone derivatives, see: Fouda *et al.* (2007 ▶); Qu *et al.* (2011 ▶); van der Star *et al.* (2012 ▶). For the use of metal(I) complexes of phosphine ligands as precursors for the preparation of mixed-ligand complexes, see: Nawaz *et al.* (2011 ▶); Pakawatchai *et al.* (2012 ▶). For a related structure, see: Wattanakanjana *et al.* (2013 ▶).
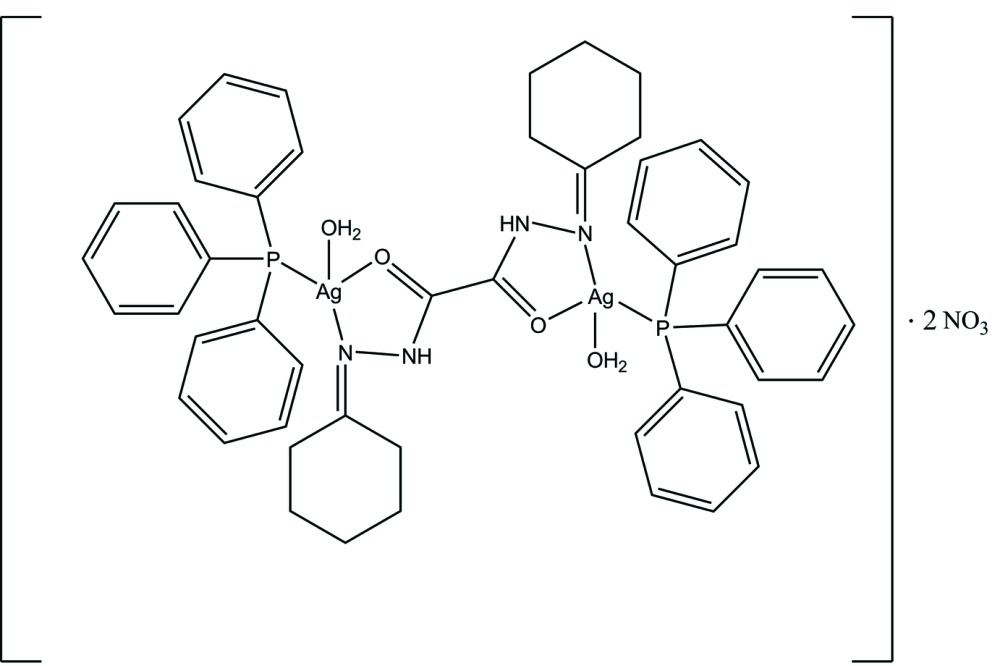


## Experimental   

### 

#### Crystal data  

[Ag_2_(C_14_H_22_N_4_O_2_)(C_18_H_15_P)_2_(H_2_O)_2_](NO_3_)_2_
*M* *_r_* = 1178.68
Triclinic, 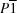

*a* = 9.0903 (8) Å
*b* = 9.5730 (8) Å
*c* = 15.2638 (13) Å
α = 74.617 (1)°
β = 83.676 (1)°
γ = 77.091 (1)°
*V* = 1246.49 (18) Å^3^
*Z* = 1
Mo *K*α radiation
μ = 0.91 mm^−1^
*T* = 100 K
0.42 × 0.38 × 0.10 mm

#### Data collection  

Bruker SMART APEX CCD diffractometer
Absorption correction: multi-scan (*SADABS*; Bruker, 2012 ▶) *T*_min_ = 0.624, *T*_max_ = 0.746
29613 measured reflections
7621 independent reflections
7076 reflections with *I* > 2σ(*I*)
*R*_int_ = 0.032

#### Refinement  

*R*[*F* ^2^ > 2σ(*F* ^2^)] = 0.028
*wR*(*F* ^2^) = 0.072
*S* = 1.02
7621 reflections
313 parameters
H-atom parameters constrained
Δρ_max_ = 1.50 e Å^−3^
Δρ_min_ = −0.54 e Å^−3^

### 

Data collection: *APEX2* (Bruker, 2012 ▶); cell refinement: *SAINT* (Bruker, 2012 ▶); data reduction: *SAINT*; program(s) used to solve structure: *SHELXS97* (Sheldrick, 2008 ▶); program(s) used to refine structure: *SHELXL2012* (Sheldrick, 2008 ▶), *SHELXLE* (Hübschle *et al.*, 2011 ▶); molecular graphics: *Mercury* (Macrae *et al.*, 2008 ▶); software used to prepare material for publication: *SHELXL97* and *publCIF* (Westrip, 2010 ▶).

## Supplementary Material

Crystal structure: contains datablock(s) I. DOI: 10.1107/S1600536813034454/lh5679sup1.cif

Structure factors: contains datablock(s) I. DOI: 10.1107/S1600536813034454/lh5679Isup2.hkl

CCDC reference:

Additional supporting information:  crystallographic information; 3D view; checkCIF report

## Figures and Tables

**Table 1:** Hydrogen-bond geometry (Å, °)

*D*—H⋯*A*	*D*—H	H⋯*A*	*D*⋯*A*	*D*—H⋯*A*
O2—H2*A*⋯O3	0.80	2.02	2.8103 (17)	167
O2—H2*B*⋯O3^i^	0.88	1.98	2.8684 (16)	177
N1—H1⋯O5^i^	0.88	2.16	2.8407 (19)	134
C22—H22⋯O4	0.95	2.58	3.297 (2)	133

Symmetry code: (i) 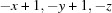.

**Fig. 1.**
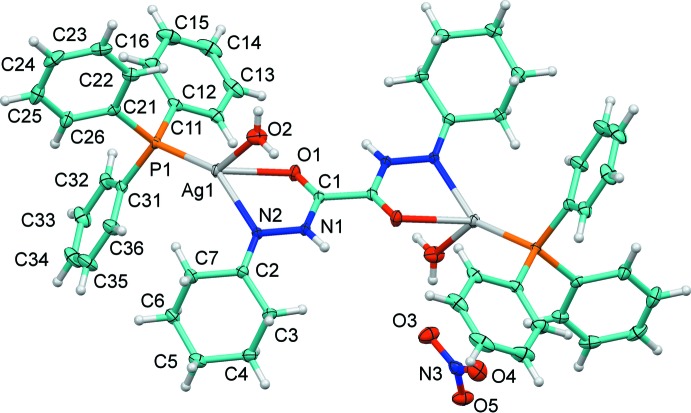
The molecular structure with displacement ellipsoids drawn at the 30% probability level. Only the symmetry unique anion is shown and the asymmetric unit labelled.

**Fig. 2.**
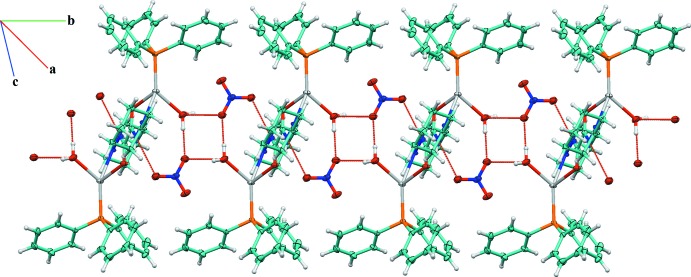
Part of the crystal structure of [{Ag(H2O)(C18H15P)}2(C14H22N4O2)]·(NO3)2 with O—H···O hydrogen bonds (red dashed lines) showing 1-D chain along [010] axis.

**Fig. 3.**
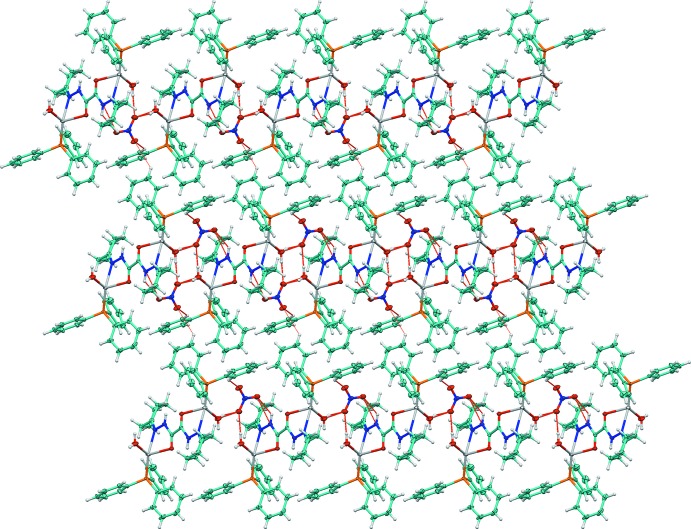
A Fragment of the 2-D network of [{Ag(H2O)(C18H15P)}2(C14H22N4O2)]·(NO3)2, showing C—H···O hydrogen bonds viewed along the a axis.

## supplementary crystallographic information

### 1. Comment

Studies of hydrazone derivatives containing nitrogen and oxygen have recently attracted considerable attention because not only are they corrosion inhibitors but it has been discovered that they are effective in different types of media (Fouda *et al.*, 2007; Qu *et al.*, 2011). They are an invaluable tool for studying mechanisms of acquired demyelination and remyelination which are histological hallmarks of multiple sclerosis (MS) (van der Star *et al.*, 2012). Silver(I) complexes of phosphine ligands have been extensively studied as precursors for preparing mixed-ligand complexes having different geometries such as mononuclear and dinuclear (Nawaz *et al.*, 2011; Pakawatchai *et al.*, 2012). Here, we report the crystal structure of the title compound.

The molecular structure of the title compound is shown in Fig. 1. The symmetry unique Ag^I^ ion is coordinated to the P atom of a triphenylphosphane ligand and the O atom of water molecule which forms the [Ag(H_2_O)(PPh_3_)] units. The bis(cyclohexanone)oxalydihydrazone ligand, located on an inversion center, acts as a bidentate bridging ligand between the two [Ag(H_2_O)(PPh_3_)] units by way of one O atom and one N atom. The Ag^I^ ion displays a distorted tetrahedral coordination. The P1—Ag1 bond length of 2.3369 (4) Å is shorter than that found in for example [Ag_2_C_l2_(CH_5_N_3_S)_2_(C_18_H_15_P)_2_], which is 2.4225 (4) Å (Wattanakanjana *et al.*, 2013). In the crystal, hydrogen bonds play a key role stabilizing a 2-D network. Intermolecular O—H···O hydrogen bonds occur where the oxygen atoms of nitrate anions serve as acceptors while H atoms of water molecules act as donors (Table 1). In addition, a pair of O—H···O hydrogen bonds form a four-membered O_2_H_2_ ring within a 1-D chain along [010] (Fig. 2). The chains are connected through weak C—H···O hydrogen bonds leading to the formation of a 2-D supramolecular network parallel to (001) as shown in Figure 3.

### 2. Experimental

Bis(cyclohexanone)oxalydihydrazone, BCO, (0.16g,0.58 mmol) was dissolved in 30 cm^3^ of methanol at 332 K. AgNO_3_ (0.10g,0.59 mmol) was added and the mixture was stirred for 3 hours. Triphenylphosphine, PPh_3_, (0.31g,1.18 mmol) was added and new reaction mixture was heated under reflux for 2 hours. The resulting clear solution was filtered off and left to evaporate at room temperature. Colorless crystals, which were deposited upon standing for 6 days, were filtered off and dried under reduced pressure.

### 3. Refinement

H atoms bonded to C and N atoms were included in calculated positions with C—H = 0.95–0.99Å, N—H = 0.88Å and refined in a riding-model approximation with U_iso_(H) = 1.2U_eq_(C,N). The H atoms of the water molecules were included 'as found' positions with 1.5U_eq_(O).

### Crystal data

**Table:**

[Ag_2_(C_14_H_22_N_4_O_2_)(C_18_H_15_P)_2_(H_2_O)_2_](NO_3_)_2_	*Z* = 1
*M**_r_* = 1178.68	*F*(000) = 602
Triclinic, *P*1	*D*_x_ = 1.570 Mg m^−^^3^
*a* = 9.0903 (8) Å	Mo *K*α radiation, λ = 0.71073 Å
*b* = 9.5730 (8) Å	Cell parameters from 6878 reflections
*c* = 15.2638 (13) Å	θ = 2.3–31.3°
α = 74.617 (1)°	µ = 0.91 mm^−^^1^
β = 83.676 (1)°	*T* = 100 K
γ = 77.091 (1)°	Plate, colourless
*V* = 1246.49 (18) Å^3^	0.42 × 0.38 × 0.10 mm

### Data collection

**Table:**

Bruker SMART APEX CCD diffractometer	7076 reflections with *I* > 2σ(*I*)
Radiation source: sealed tube	*R*_int_ = 0.032
φ and ω scans	θ_max_ = 31.6°, θ_min_ = 2.3°
Absorption correction: multi-scan (*SADABS*; Bruker, 2012)	*h* = −13→13
*T*_min_ = 0.624, *T*_max_ = 0.746	*k* = −13→13
29613 measured reflections	*l* = −22→22
7621 independent reflections	

### Refinement

**Table:**

Refinement on *F*^2^	Primary atom site location: structure-invariant direct methods
Least-squares matrix: full	Secondary atom site location: difference Fourier map
*R*[*F*^2^ > 2σ(*F*^2^)] = 0.028	Hydrogen site location: mixed
*wR*(*F*^2^) = 0.072	H-atom parameters constrained
*S* = 1.02	*w* = 1/[σ^2^(*F*_o_^2^) + (0.0427*P*)^2^ + 0.444*P*] where *P* = (*F*_o_^2^ + 2*F*_c_^2^)/3
7621 reflections	(Δ/σ)_max_ = 0.001
313 parameters	Δρ_max_ = 1.50 e Å^−^^3^
0 restraints	Δρ_min_ = −0.54 e Å^−^^3^

### Special details

**Table:**

Experimental. Reflections 0 0 1 was affected by the beam stop and was omitted from the refinement.
Geometry. All e.s.d.'s (except the e.s.d. in the dihedral angle between two l.s. planes) are estimated using the full covariance matrix. The cell e.s.d.'s are taken into account individually in the estimation of e.s.d.'s in distances, angles and torsion angles; correlations between e.s.d.'s in cell parameters are only used when they are defined by crystal symmetry. An approximate (isotropic) treatment of cell e.s.d.'s is used for estimating e.s.d.'s involving l.s. planes.

### Fractional atomic coordinates and isotropic or equivalent isotropic displacement parameters (Å^2^)

**Table:**

	*x*	*y*	*z*	*U*_iso_*/*U*_eq_	
Ag1	0.77397 (2)	0.67531 (2)	0.14033 (2)	0.01512 (4)	
P1	0.87389 (4)	0.59821 (4)	0.28403 (3)	0.01204 (7)	
O1	0.58233 (10)	0.91020 (10)	0.10413 (6)	0.0182 (2)	
O2	0.60176 (10)	0.57786 (10)	0.07530 (6)	0.0278 (3)	
H2A	0.5453	0.5216	0.0951	0.042*	
H2B	0.5935	0.5990	0.0158	0.042*	
O3	0.41980 (18)	0.36454 (16)	0.11758 (9)	0.0311 (3)	
O4	0.46973 (17)	0.30464 (18)	0.26020 (10)	0.0327 (3)	
O5	0.37588 (17)	0.15741 (16)	0.20689 (9)	0.0289 (3)	
N1	0.67258 (14)	0.90320 (14)	−0.04115 (9)	0.0146 (2)	
H1	0.6597	0.9387	−0.0998	0.017*	
N2	0.80176 (14)	0.79893 (14)	−0.00875 (9)	0.0144 (2)	
N3	0.42173 (16)	0.27494 (17)	0.19571 (10)	0.0211 (3)	
C1	0.56953 (16)	0.94638 (16)	0.02131 (10)	0.0132 (3)	
C2	0.91980 (17)	0.79182 (17)	−0.06359 (10)	0.0160 (3)	
C3	0.93888 (19)	0.88935 (19)	−0.15685 (11)	0.0203 (3)	
H3A	0.8466	0.9672	−0.1707	0.024*	
H3B	0.9540	0.8301	−0.2025	0.024*	
C4	1.0763 (2)	0.96139 (19)	−0.16206 (12)	0.0220 (3)	
H4A	1.0959	1.0152	−0.2258	0.026*	
H4B	1.0528	1.0343	−0.1245	0.026*	
C5	1.21787 (19)	0.8478 (2)	−0.12903 (12)	0.0222 (3)	
H5A	1.2474	0.7802	−0.1699	0.027*	
H5B	1.3020	0.8989	−0.1309	0.027*	
C6	1.19021 (19)	0.75881 (19)	−0.03247 (12)	0.0210 (3)	
H6A	1.1659	0.8255	0.0091	0.025*	
H6B	1.2827	0.6846	−0.0123	0.025*	
C7	1.05827 (18)	0.68022 (18)	−0.02872 (11)	0.0185 (3)	
H7A	1.0861	0.6074	−0.0663	0.022*	
H7B	1.0371	0.6264	0.0348	0.022*	
C11	0.74414 (17)	0.67524 (18)	0.36679 (11)	0.0163 (3)	
C12	0.66381 (18)	0.82024 (19)	0.33780 (12)	0.0204 (3)	
H12	0.6780	0.8747	0.2768	0.024*	
C13	0.5628 (2)	0.8854 (2)	0.39823 (15)	0.0289 (4)	
H13	0.5084	0.9842	0.3786	0.035*	
C14	0.5425 (2)	0.8049 (3)	0.48700 (15)	0.0347 (5)	
H14	0.4741	0.8492	0.5284	0.042*	
C15	0.6211 (2)	0.6599 (3)	0.51627 (14)	0.0338 (4)	
H15	0.6058	0.6054	0.5772	0.041*	
C16	0.7224 (2)	0.5946 (2)	0.45616 (12)	0.0244 (3)	
H16	0.7765	0.4957	0.4759	0.029*	
C21	0.91018 (18)	0.39933 (16)	0.33027 (10)	0.0146 (3)	
C22	0.7932 (2)	0.32645 (19)	0.32916 (11)	0.0201 (3)	
H22	0.6984	0.3814	0.3067	0.024*	
C23	0.8160 (2)	0.1736 (2)	0.36097 (12)	0.0262 (4)	
H23	0.7362	0.1240	0.3613	0.031*	
C24	0.9551 (3)	0.0933 (2)	0.39231 (13)	0.0297 (4)	
H24	0.9703	−0.0113	0.4139	0.036*	
C25	1.0718 (3)	0.1643 (2)	0.39229 (14)	0.0309 (4)	
H25	1.1671	0.1084	0.4132	0.037*	
C26	1.0499 (2)	0.31784 (19)	0.36159 (12)	0.0222 (3)	
H26	1.1299	0.3667	0.3620	0.027*	
C31	1.05027 (17)	0.65300 (16)	0.29052 (11)	0.0152 (3)	
C32	1.07908 (19)	0.70550 (18)	0.36258 (12)	0.0204 (3)	
H32	1.0065	0.7101	0.4120	0.025*	
C33	1.2145 (2)	0.7513 (2)	0.36192 (15)	0.0296 (4)	
H33	1.2338	0.7877	0.4108	0.036*	
C34	1.3209 (2)	0.7438 (2)	0.29016 (16)	0.0337 (4)	
H34	1.4121	0.7769	0.2895	0.040*	
C35	1.2953 (2)	0.6887 (3)	0.21933 (15)	0.0322 (4)	
H35	1.3696	0.6816	0.1709	0.039*	
C36	1.1601 (2)	0.6437 (2)	0.21948 (13)	0.0243 (3)	
H36	1.1421	0.6062	0.1708	0.029*	

### Atomic displacement parameters (Å^2^)

**Table:**

	*U*^11^	*U*^22^	*U*^33^	*U*^12^	*U*^13^	*U*^23^
Ag1	0.01432 (6)	0.01811 (6)	0.01070 (6)	0.00005 (4)	−0.00308 (4)	−0.00155 (4)
P1	0.01111 (16)	0.01418 (16)	0.01055 (16)	−0.00195 (13)	−0.00170 (12)	−0.00267 (13)
O1	0.0195 (5)	0.0194 (5)	0.0129 (5)	0.0032 (4)	−0.0034 (4)	−0.0040 (4)
O2	0.0328 (7)	0.0370 (7)	0.0208 (6)	−0.0188 (6)	−0.0022 (5)	−0.0096 (5)
O3	0.0411 (8)	0.0359 (7)	0.0194 (6)	−0.0171 (6)	−0.0039 (6)	−0.0038 (5)
O4	0.0329 (7)	0.0479 (9)	0.0250 (7)	−0.0109 (6)	−0.0062 (6)	−0.0180 (6)
O5	0.0355 (7)	0.0358 (7)	0.0203 (6)	−0.0179 (6)	0.0022 (5)	−0.0078 (5)
N1	0.0128 (6)	0.0159 (6)	0.0118 (5)	0.0001 (5)	−0.0018 (4)	0.0000 (5)
N2	0.0106 (5)	0.0161 (6)	0.0137 (6)	0.0000 (4)	−0.0011 (4)	−0.0010 (5)
N3	0.0161 (6)	0.0308 (7)	0.0187 (6)	−0.0047 (6)	0.0005 (5)	−0.0107 (6)
C1	0.0121 (6)	0.0127 (6)	0.0146 (6)	−0.0020 (5)	−0.0030 (5)	−0.0023 (5)
C2	0.0140 (7)	0.0186 (7)	0.0151 (7)	−0.0040 (5)	−0.0003 (5)	−0.0034 (5)
C3	0.0171 (7)	0.0265 (8)	0.0140 (7)	−0.0055 (6)	0.0016 (5)	0.0005 (6)
C4	0.0216 (8)	0.0230 (8)	0.0205 (8)	−0.0075 (6)	0.0036 (6)	−0.0033 (6)
C5	0.0177 (7)	0.0277 (8)	0.0237 (8)	−0.0084 (6)	0.0026 (6)	−0.0092 (7)
C6	0.0151 (7)	0.0267 (8)	0.0219 (8)	−0.0028 (6)	−0.0003 (6)	−0.0088 (6)
C7	0.0139 (7)	0.0193 (7)	0.0203 (7)	−0.0008 (6)	−0.0003 (6)	−0.0041 (6)
C11	0.0124 (6)	0.0231 (7)	0.0168 (7)	−0.0053 (6)	0.0005 (5)	−0.0095 (6)
C12	0.0148 (7)	0.0222 (7)	0.0286 (8)	−0.0055 (6)	0.0001 (6)	−0.0131 (7)
C13	0.0178 (8)	0.0323 (9)	0.0458 (11)	−0.0070 (7)	0.0044 (7)	−0.0266 (9)
C14	0.0253 (9)	0.0514 (12)	0.0426 (11)	−0.0163 (9)	0.0126 (8)	−0.0367 (10)
C15	0.0325 (10)	0.0552 (13)	0.0228 (9)	−0.0190 (9)	0.0096 (7)	−0.0213 (9)
C16	0.0246 (8)	0.0339 (9)	0.0160 (7)	−0.0081 (7)	0.0016 (6)	−0.0079 (7)
C21	0.0181 (7)	0.0148 (6)	0.0109 (6)	−0.0044 (5)	−0.0003 (5)	−0.0022 (5)
C22	0.0209 (8)	0.0215 (7)	0.0201 (7)	−0.0086 (6)	0.0028 (6)	−0.0069 (6)
C23	0.0357 (10)	0.0241 (8)	0.0231 (8)	−0.0164 (7)	0.0103 (7)	−0.0094 (7)
C24	0.0493 (12)	0.0160 (7)	0.0212 (8)	−0.0078 (8)	0.0020 (8)	−0.0008 (6)
C25	0.0383 (11)	0.0196 (8)	0.0289 (9)	0.0019 (7)	−0.0101 (8)	0.0010 (7)
C26	0.0249 (8)	0.0183 (7)	0.0213 (8)	−0.0020 (6)	−0.0074 (6)	−0.0005 (6)
C31	0.0128 (6)	0.0141 (6)	0.0180 (7)	−0.0027 (5)	−0.0029 (5)	−0.0021 (5)
C32	0.0175 (7)	0.0209 (7)	0.0248 (8)	−0.0042 (6)	−0.0044 (6)	−0.0073 (6)
C33	0.0219 (8)	0.0336 (10)	0.0408 (11)	−0.0080 (7)	−0.0083 (8)	−0.0173 (8)
C34	0.0171 (8)	0.0388 (11)	0.0508 (13)	−0.0101 (8)	−0.0037 (8)	−0.0163 (9)
C35	0.0167 (8)	0.0450 (11)	0.0377 (11)	−0.0099 (8)	0.0055 (7)	−0.0147 (9)
C36	0.0175 (8)	0.0337 (9)	0.0253 (8)	−0.0082 (7)	0.0026 (6)	−0.0123 (7)

### Geometric parameters (Å, º)

**Table:**

Ag1—N2	2.2849 (13)	C11—C16	1.395 (2)
Ag1—P1	2.3369 (4)	C11—C12	1.396 (2)
Ag1—O2	2.4068	C12—C13	1.395 (2)
Ag1—O1	2.4898 (9)	C12—H12	0.9500
P1—C21	1.8137 (15)	C13—C14	1.385 (3)
P1—C31	1.8149 (16)	C13—H13	0.9500
P1—C11	1.8187 (16)	C14—C15	1.391 (3)
O1—C1	1.2304 (17)	C14—H14	0.9500
O2—H2A	0.8048	C15—C16	1.393 (3)
O2—H2B	0.8848	C15—H15	0.9500
O3—N3	1.270 (2)	C16—H16	0.9500
O4—N3	1.2404 (19)	C21—C26	1.394 (2)
O5—N3	1.249 (2)	C21—C22	1.400 (2)
N1—C1	1.340 (2)	C22—C23	1.389 (2)
N1—N2	1.4008 (17)	C22—H22	0.9500
N1—H1	0.8800	C23—C24	1.387 (3)
N2—C2	1.287 (2)	C23—H23	0.9500
C1—C1^i^	1.524 (3)	C24—C25	1.382 (3)
C2—C3	1.497 (2)	C24—H24	0.9500
C2—C7	1.501 (2)	C25—C26	1.394 (2)
C3—C4	1.542 (2)	C25—H25	0.9500
C3—H3A	0.9900	C26—H26	0.9500
C3—H3B	0.9900	C31—C32	1.395 (2)
C4—C5	1.523 (2)	C31—C36	1.398 (2)
C4—H4A	0.9900	C32—C33	1.394 (2)
C4—H4B	0.9900	C32—H32	0.9500
C5—C6	1.519 (2)	C33—C34	1.385 (3)
C5—H5A	0.9900	C33—H33	0.9500
C5—H5B	0.9900	C34—C35	1.384 (3)
C6—C7	1.541 (2)	C34—H34	0.9500
C6—H6A	0.9900	C35—C36	1.390 (3)
C6—H6B	0.9900	C35—H35	0.9500
C7—H7A	0.9900	C36—H36	0.9500
C7—H7B	0.9900		
			
N2—Ag1—P1	146.83 (3)	C2—C7—H7B	109.7
N2—Ag1—O2	80.48 (4)	C6—C7—H7B	109.7
P1—Ag1—O2	130.70 (2)	H7A—C7—H7B	108.2
N2—Ag1—O1	69.04 (4)	C16—C11—C12	120.00 (15)
P1—Ag1—O1	118.43 (2)	C16—C11—P1	122.44 (13)
O2—Ag1—O1	84.40 (3)	C12—C11—P1	117.56 (12)
C21—P1—C31	105.26 (7)	C13—C12—C11	120.17 (17)
C21—P1—C11	105.67 (7)	C13—C12—H12	119.9
C31—P1—C11	104.89 (7)	C11—C12—H12	119.9
C21—P1—Ag1	113.89 (5)	C14—C13—C12	119.49 (19)
C31—P1—Ag1	115.29 (5)	C14—C13—H13	120.3
C11—P1—Ag1	110.99 (5)	C12—C13—H13	120.3
C1—O1—Ag1	107.71 (8)	C13—C14—C15	120.72 (17)
Ag1—O2—H2A	135.1	C13—C14—H14	119.6
Ag1—O2—H2B	121.5	C15—C14—H14	119.6
H2A—O2—H2B	103.3	C14—C15—C16	119.94 (19)
C1—N1—N2	116.91 (12)	C14—C15—H15	120.0
C1—N1—H1	121.5	C16—C15—H15	120.0
N2—N1—H1	121.5	C15—C16—C11	119.67 (19)
C2—N2—N1	117.05 (13)	C15—C16—H16	120.2
C2—N2—Ag1	129.26 (11)	C11—C16—H16	120.2
N1—N2—Ag1	113.55 (9)	C26—C21—C22	119.82 (15)
O4—N3—O5	120.55 (16)	C26—C21—P1	122.88 (12)
O4—N3—O3	119.69 (16)	C22—C21—P1	117.21 (12)
O5—N3—O3	119.76 (14)	C23—C22—C21	119.81 (17)
O1—C1—N1	125.99 (13)	C23—C22—H22	120.1
O1—C1—C1^i^	121.54 (17)	C21—C22—H22	120.1
N1—C1—C1^i^	112.43 (16)	C24—C23—C22	120.06 (17)
N2—C2—C3	127.37 (15)	C24—C23—H23	120.0
N2—C2—C7	117.00 (14)	C22—C23—H23	120.0
C3—C2—C7	115.50 (13)	C25—C24—C23	120.41 (16)
C2—C3—C4	109.85 (14)	C25—C24—H24	119.8
C2—C3—H3A	109.7	C23—C24—H24	119.8
C4—C3—H3A	109.7	C24—C25—C26	120.14 (18)
C2—C3—H3B	109.7	C24—C25—H25	119.9
C4—C3—H3B	109.7	C26—C25—H25	119.9
H3A—C3—H3B	108.2	C21—C26—C25	119.75 (17)
C5—C4—C3	112.15 (14)	C21—C26—H26	120.1
C5—C4—H4A	109.2	C25—C26—H26	120.1
C3—C4—H4A	109.2	C32—C31—C36	119.17 (15)
C5—C4—H4B	109.2	C32—C31—P1	122.66 (12)
C3—C4—H4B	109.2	C36—C31—P1	118.16 (12)
H4A—C4—H4B	107.9	C33—C32—C31	119.95 (17)
C6—C5—C4	110.70 (14)	C33—C32—H32	120.0
C6—C5—H5A	109.5	C31—C32—H32	120.0
C4—C5—H5A	109.5	C34—C33—C32	120.11 (18)
C6—C5—H5B	109.5	C34—C33—H33	119.9
C4—C5—H5B	109.5	C32—C33—H33	119.9
H5A—C5—H5B	108.1	C35—C34—C33	120.54 (17)
C5—C6—C7	109.82 (14)	C35—C34—H34	119.7
C5—C6—H6A	109.7	C33—C34—H34	119.7
C7—C6—H6A	109.7	C34—C35—C36	119.52 (18)
C5—C6—H6B	109.7	C34—C35—H35	120.2
C7—C6—H6B	109.7	C36—C35—H35	120.2
H6A—C6—H6B	108.2	C35—C36—C31	120.69 (17)
C2—C7—C6	109.86 (13)	C35—C36—H36	119.7
C2—C7—H7A	109.7	C31—C36—H36	119.7
C6—C7—H7A	109.7		
			
C1—N1—N2—C2	−158.29 (14)	C12—C11—C16—C15	−0.4 (3)
C1—N1—N2—Ag1	17.79 (16)	P1—C11—C16—C15	−179.89 (14)
Ag1—O1—C1—N1	−22.20 (17)	C31—P1—C21—C26	−0.36 (16)
Ag1—O1—C1—C1^i^	160.03 (15)	C11—P1—C21—C26	−111.04 (14)
N2—N1—C1—O1	5.0 (2)	Ag1—P1—C21—C26	126.88 (13)
N2—N1—C1—C1^i^	−177.05 (14)	C31—P1—C21—C22	−176.91 (12)
N1—N2—C2—C3	4.3 (2)	C11—P1—C21—C22	72.41 (13)
Ag1—N2—C2—C3	−171.05 (12)	Ag1—P1—C21—C22	−49.67 (13)
N1—N2—C2—C7	179.89 (13)	C26—C21—C22—C23	1.3 (2)
Ag1—N2—C2—C7	4.5 (2)	P1—C21—C22—C23	177.95 (13)
N2—C2—C3—C4	123.63 (18)	C21—C22—C23—C24	−1.1 (3)
C7—C2—C3—C4	−52.01 (19)	C22—C23—C24—C25	0.1 (3)
C2—C3—C4—C5	51.65 (19)	C23—C24—C25—C26	0.7 (3)
C3—C4—C5—C6	−56.69 (19)	C22—C21—C26—C25	−0.5 (3)
C4—C5—C6—C7	58.54 (18)	P1—C21—C26—C25	−176.97 (14)
N2—C2—C7—C6	−121.04 (16)	C24—C25—C26—C21	−0.5 (3)
C3—C2—C7—C6	55.07 (19)	C21—P1—C31—C32	−95.87 (14)
C5—C6—C7—C2	−56.55 (18)	C11—P1—C31—C32	15.37 (15)
C21—P1—C11—C16	17.38 (16)	Ag1—P1—C31—C32	137.74 (12)
C31—P1—C11—C16	−93.57 (15)	C21—P1—C31—C36	85.04 (14)
Ag1—P1—C11—C16	141.31 (13)	C11—P1—C31—C36	−163.72 (13)
C21—P1—C11—C12	−162.09 (12)	Ag1—P1—C31—C36	−41.35 (15)
C31—P1—C11—C12	86.96 (13)	C36—C31—C32—C33	1.6 (3)
Ag1—P1—C11—C12	−38.16 (13)	P1—C31—C32—C33	−177.50 (14)
C16—C11—C12—C13	0.6 (2)	C31—C32—C33—C34	−0.4 (3)
P1—C11—C12—C13	−179.91 (13)	C32—C33—C34—C35	−1.1 (3)
C11—C12—C13—C14	−0.2 (3)	C33—C34—C35—C36	1.5 (3)
C12—C13—C14—C15	−0.3 (3)	C34—C35—C36—C31	−0.3 (3)
C13—C14—C15—C16	0.5 (3)	C32—C31—C36—C35	−1.2 (3)
C14—C15—C16—C11	−0.1 (3)	P1—C31—C36—C35	177.89 (16)

Symmetry code: (i) −*x*+1, −*y*+2, −*z*.

### Hydrogen-bond geometry (Å, º)

**Table:**

*D*—H···*A*	*D*—H	H···*A*	*D*···*A*	*D*—H···*A*
O2—H2*A*···O3	0.80	2.02	2.8103 (17)	167
O2—H2*B*···O3^ii^	0.88	1.98	2.8684 (16)	177
N1—H1···O5^ii^	0.88	2.16	2.8407 (19)	134
C22—H22···O4	0.95	2.58	3.297 (2)	133

Symmetry code: (ii) −*x*+1, −*y*+1, −*z*.
